# Soldiers' Dental Aid Fund

**Published:** 1915-01-02

**Authors:** Betty Conyngham, Ada Elizabeth Fletcher

**Affiliations:** Hon Secretary.; Operating Hon. Secretary.


### THE EDITOR'S LETTER-BOX.

### Soldiers' Dental Aid Fund.

*To the Editor of* The Hospital.

Sir,—Lord Kitchener is still appealing for soldiers. There are a number of men willing who cannot take their place in the field until their teeth are in order. The Royal Dental Hospital and the National Dental Hospital and other hospitals have done and are doing a grand work for the soldier, but funds are now urgently needed to provide them with proper teeth.

Many an able-bodied man is waiting and worrying for these teeth to enable him to go forward and become a fit and proper soldier; we know this because the men are still applying to the hospitals. Will not those of us particularly who cannot have the glorious privilege of actively serving our King and country, make it possible for these soldiers to do so by generously subscribing to this fund?

The Lord Mayor is interested in the fund. The Duke of Norfolk, K.G., is warmly supporting it; we have the approval of Lord Rothschild, as President of the Royal Dental Hospital, and Viscount Portman, as President of the National Dental Hospital.

Offices have been kindly lent at 36 Leicester Square, W.C., where all communications and cheques (made payable to the Hon. Treasurer) should be addressed to the Operating Hon. Secretary, Ada Elizabeth Fletcher.—Yours faithfully,

Betty Conyngham,
Hon Secretary.
Ada Elizabeth Fletcher,
Operating Hon. Secretary.

